# The double-edged sword effect of empowering leadership on illegitimate tasks: role boundary redefinition and individual differences

**DOI:** 10.3389/fpsyg.2025.1702471

**Published:** 2026-01-12

**Authors:** Qin Yang, Zhijun Jin, Jing Shao, Fang Guo, Xujuan Huang, Yin Wenjie, Yu Wang

**Affiliations:** Department of Psychology, School of Education, China University of Geosciences, Wuhan, China

**Keywords:** empowering leadership, illegitimate tasks, perception of illegitimate tasks, role stress, work passion

## Abstract

In contemporary organizations, employees often encounter illegitimate tasks, which violate their professional role expectations and undermine their occupational identity. Drawing on role theory and the Job Demands–Resources (JD-R) model, this study investigates how empowering leadership influences perceptions of illegitimate tasks through role boundary redefinition. Analysis of survey data from 354 corporate employees reveals that empowering leadership reduces perceptions of illegitimate tasks by enhancing work passion, while increasing them through elevated role stress. Furthermore, regulatory focus moderates these relationships: promotion focus strengthens the link between empowering leadership and work passion, while prevention focus amplifies the association between empowering leadership and role stress. The study underscores that perceptions of illegitimate tasks are subjective and shaped by the dynamic renegotiation of role boundaries initiated by empowering leadership, with individual differences, particularly regulatory focus, influencing whether employees perceive these tasks as growth opportunities or burdens that exceed their role expectations.

## 1 Introduction

In contemporary organizations, employees are formally expected to perform duties that correspond to their professional roles (Khumalo and Olaleye, 2025). Role theory conceptualizes such roles as bundles of behavioral expectations attached to positions, communicated by “role senders” such as the organization, leaders and colleagues (Katz and Kahn, 1978). In practice, however, employees are often required to carry out tasks they experience as unnecessary, inappropriate or incompatible with their occupational identity. These illegitimate tasks are defined as assignments that violate professional role expectations and threaten employees’ sense of occupational worth (Semmer et al., 2007; Semmer et al., 2010). Early studies highlight the ubiquity of this phenomenon. For example, a study involving 159 employees who listed over 3,500 daily tasks found that approximately one-third of these tasks were perceived as illegitimate, with 65% of secondary tasks considered inappropriate (Jacobshagen, 2006). These findings demonstrate that illegitimate tasks are not isolated incidents but a pervasive challenge in organizational life.

While research has extensively documented the detrimental consequences of illegitimate tasks, such as impaired self-esteem (Semmer et al., 2015; Semmer et al., 2019), diminished work meaningfulness (Eatough et al., 2016), burnout (García Johnson and Otto, 2020), and procrastination (Zou et al., 2023), less is known about their antecedents. Existing research has largely focused on task characteristics, such as performance pressure (Ma and Peng, 2019), or organizational structures, such as organizational size (Fältén et al., 2024). However, perceptions of illegitimacy are fundamentally cognitive rather than objective (Semmer et al., 2015), shaped by employees’ judgments of “what is part of one’s role” (Zhou et al., 2020). This calls for research that moves beyond static task features and explores the dynamic factors that shape how employees’ role boundaries are constructed and renegotiated over time.

Leadership plays a critical role in this process, significantly influencing how employees interpret their responsibilities. As representatives of the organization, leaders define and redefine employees’ roles, which in turn affects their perception of task legitimacy (Morrison, 1994; Paterson and Huang, 2018). Among various leadership styles, empowering leadership stands out due to its paradoxical nature. While it provides autonomy, trust, and meaning (Ahearne et al., 2005), it simultaneously increases responsibilities and job complexity (Lee et al., 2018). We argue that this paradox arises because empowering leadership functions not only as a source of resources or demands but also as a process of role-boundary redefinition. Through delegating authority and assigning new tasks, leaders reshape employees’ understanding of what constitutes their role. When this expanded role aligns with employees’ expectations, empowerment is experienced as an enriching opportunity. However, when it exceeds expectations, it is perceived as an unwelcome intrusion into their professional identity.

This study extends role theory by showing that leaders do not just clarify role expectations; they actively renegotiate them. The outcomes of this renegotiation depend on how employees perceive the expanded role boundaries. We propose that these perceptions are influenced by the Job Demands–Resources (JD-R) model (Bakker and Demerouti, 2017), which provides a framework for understanding how empowering leadership impacts illegitimate task perceptions. According to the JD-R model, leadership behaviors can offer both resources (e.g., autonomy, meaningful work) and introduce demands (e.g., increased responsibility, role complexity). Empowering leadership triggers two distinct pathways: a resource-gaining pathway through work passion, where employees embrace new tasks as growth opportunities, and a resource-depleting pathway through role stress, where employees view new tasks as excessive demands that exceed their capacity, increasing perceptions of illegitimacy.

While both pathways are present, the impact of empowering leadership is further shaped by individual differences. Specifically, employees’ regulatory focus, whether they are promotion-focused (driven by growth and achievement) or prevention-focused (concerned with safety and obligations), moderates how they perceive and respond to role redefinition. Promotion-focused employees are more likely to view expanded roles as opportunities for development, fostering work passion and reducing perceptions of illegitimacy (Ahearne et al., 2005; Lanaj et al., 2012). In contrast, prevention-focused employees are more likely to experience role expansion as a threat, increasing role stress and heightening perceptions of illegitimate tasks (Higgins, 1997; Lanaj et al., 2012).

**Figure 1:**
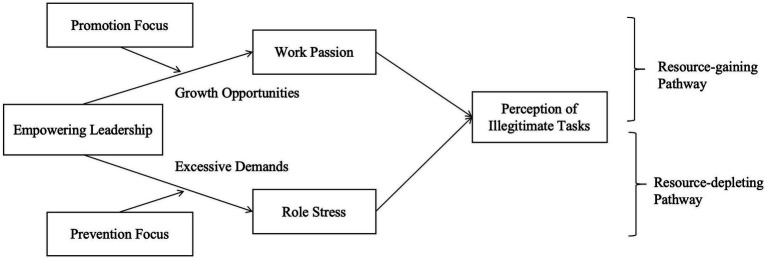
The conceptual model based on the Job Demands–Resources (JD-R) theory.

This study provides several key theoretical contributions. First, it presents a role-boundary redefinition perspective on empowering leadership, showing that it reshapes employees’ role boundaries. This shift in perspective moves beyond viewing empowerment merely as a source of resources or demands, emphasizing it as a dynamic process that redefines roles and, consequently, affects perceptions of legitimacy. Second, this study emphasizes the cognitive interpretation of illegitimate tasks, demonstrating that these perceptions arise from employees’ cognitive appraisals of their roles. By integrating the JD-R model, we show that leadership behavior influences both positive and negative pathways, either fostering engagement through work passion or causing role stress through excessive demands. Finally, this study clarifies why empowering leadership has heterogeneous effects. By highlighting the role of individual differences, specifically regulatory focus, it explains why some employees perceive new responsibilities as opportunities while others view them as burdens. This framework deepens our understanding of when empowering leadership is perceived as an opportunity and when it may become a burden, offering a more nuanced view of leadership’s effects on task legitimacy (Figure 1).

## 2 Theoretical background and hypotheses

### 2.1 Empowering leadership as a catalyst for role boundary redefinition: a JD-R perspective

The perception of illegitimate tasks is fundamentally a cognitive judgment situated within the interplay between individual role identity and organizational expectations. While illegitimate tasks are defined as assignments that violate professional role boundaries (Semmer et al., 2010), their recognition stems not from objective task features but from a subjective appraisal of role-task fit (Semmer et al., 2015). This appraisal occurs within a social context where role boundaries are subject to potential renegotiation (Katz and Kahn, 1978). The inherent ambiguity in role boundaries (Rizzo et al., 1970) creates a psychological space wherein leadership behaviors critically influence how employees define their “prescribed duties.”

Empowering leadership, characterized by delegating authority, sharing information, and encouraging self-direction (Ahearne et al., 2005), constitutes a potent, formal intervention in this process. We conceptualize empowering leadership as a trigger for role boundary redefinition. It actively puts forward a redefinition proposal by expanding an employee’s decision-making scope and task domain. As framed by the Job Demands–Resources (JD-R) model, this proposal is inherently paradoxical (Bakker and Demerouti, 2017). Empowering leadership simultaneously delivers critical job resources (e.g., autonomy, trust, meaningfulness) that can facilitate growth, and introduces new job demands (e.g., heightened responsibility, complexity, and ambiguity) that may deplete energy (Li et al., 2024).

Consequently, the impact of empowering leadership on illegitimate task perception is posited as the outcome of an employee’s cognitive and affective evaluation of this redefinition proposal. The JD-R model’s dual pathways provide the overarching explanatory mechanism: the resource-gain pathway suggests resources can foster positive integration of new duties, potentially reducing illegitimacy perceptions, whereas the health-impairment pathway suggests that excessive demands can trigger defensive exclusion of new tasks, heightening perceptions of illegitimacy (Lee and Jo, 2023). The following sections delineate the specific mediators embodying these two pathways.

### 2.2 The mediating role of work passion: the resource-gain pathway

Work passion, defined as a strong, affect-laden inclination toward one’s work that is internalized into identity (Vallerand et al., 2003), represents a key psychological outcome of the resource-gain pathway. Empowering leadership fosters work passion by providing essential resources that satisfy basic psychological needs (Song and Chen, 2021; Ye et al., 2022; Li et al., 2024). The autonomy and trust inherent in empowerment directly support needs for competence and self-determination (Deci and Ryan, 2000), while the enhanced meaningfulness of work contributes to a sense of purpose (Ho et al., 2018). According to the JD-R model’s motivational process, such resources boost intrinsic motivation (Bakker and Demerouti, 2007; Bakker et al., 2005; Lee and Jo, 2023), facilitating the internalization of work activities, which is a core process in passion development (Vallerand et al., 2003). When employees internalize empowered roles as aligned with their self-concept, work passion is enhanced.

*H1a*: Empowering leadership is positively related to work passion.

Work passion, in turn, transforms how employees perceive and incorporate new tasks. Passionate employees possess more abundant psychological resources and view their work role through an integrative, opportunity-focused lens (Ho et al., 2018; Rai et al., 2024). They are more likely to interpret challenges, including newly assigned responsibilities from empowerment (Arshad et al., 2022), as avenues for self-realization and mastery (Perrewé et al., 2014), and to construe additional demands as opportunities for growth rather than as unfair burdens (Bakker et al., 2005; Peng and Wang, 2018). This positive cognitive framing encourages the proactive integration of new tasks into their existing role identity, thereby minimizing the perceived discrepancy between the task and their professional boundaries. Consequently, tasks are less likely to be judged as illegitimate.

*H1b*: Work passion is negatively related to perceptions of illegitimate tasks.

Integrating H1a and H1b, we propose a mediating mechanism whereby empowering leadership utilizes its resource provisions to cultivate work passion, which subsequently promotes an expansive role identity that assimilates new tasks, reducing illegitimacy perceptions.

*H1*: Work passion mediates the relationship between empowering leadership and perceptions of illegitimate tasks, such that empowering leadership reduces such perceptions by increasing work passion.

### 2.3 The mediating role of role stress: the resource-depleting pathway

Conversely, the same behaviors that define empowering leadership can also heighten role stress, a state comprising role ambiguity, overload, and conflict (Kahn et al., 1964; Roy et al., 1965), via the resource-depleting pathway. Within the Job Demands–Resources (JD-R) model, such role stress reflects heightened job demands that require sustained physical, cognitive, and emotional effort, and therefore consume employees’ limited resources (Bakker and Demerouti, 2007). While granting autonomy, the delegation of authority often expands task scope without providing immediate clarity, potentially fostering role ambiguity as employees grapple with ill-defined new responsibilities (Lorinkova et al., 2013). The accompanying increase in responsibility and performance expectations can contribute to role overload, particularly when expectations are high and resources are insufficient (Dennerlein and Kirkman, 2022; Wang et al., 2021). Furthermore, newly empowered duties may clash with pre-existing priorities, generating role conflict. Together, these elements constitute significant job demands that require sustained cognitive and emotional effort and can erode employees’ capacity to cope effectively.

*H2a*: Empowering leadership is positively related to role stress.

According to the JD-R model, chronic job demands deplete an individual’s personal resources (Bakker and Demerouti, 2017). Role stress is a direct manifestation of this resource drain and has been shown to impair performance and psychological well-being (Jin et al., 2014; Tang et al., 2023). When experiencing role stress, employees enter a resource-conservation state, adopting a defensive posture toward their remaining energy and existing role boundaries (Hobfoll, 1989). In this state, additional tasks, particularly those stemming from the empowering acts that initially created the stress, are more likely to be appraised as intrusive threats. To protect their depleted resources and defend their current role schema, employees cognitively reject these tasks by categorizing them as illegitimate, thereby justifying their exclusion from the role domain.

*H2b*: Role stress is positively related to perceptions of illegitimate tasks.

Thus, the motivational intent of empowering leadership can, when perceived as a source of excessive or poorly defined demands, generate role stress. This stress depletes resources and triggers a defensive narrowing of role boundaries, increasing the likelihood of labeling new tasks as illegitimate.

*H2*: Role stress mediates the relationship between empowering leadership and perceptions of illegitimate tasks, such that empowering leadership increases such perceptions by elevating role stress.

### 2.4 The moderating role of regulatory focus

The divergent pathways outlined above are not equally salient for all employees. We propose that an individual’s regulatory focus (Higgins, 1997; Higgins and Pinelli, 2020) acts as a critical cognitive filter, determining how the role redefinition proposal initiated by empowering leadership is appraised and which subsequent pathway is amplified. Employees with a dominant promotion focus, who are attuned to growth, aspirations, and gains (Lanaj et al., 2012; Yan et al., 2024), are more likely to perceive the resources (e.g., autonomy, opportunity) in empowering leadership (Bakker and Demerouti, 2017; Ye et al., 2022). This positive appraisal makes them more susceptible to the resource-gain pathway, strengthening the link between empowering leadership and work passion (Vallerand et al., 2003; Ho et al., 2018).

Conversely, employees with a dominant prevention focus, who are vigilant about safety, obligations, and potential losses (Lanaj et al., 2012; Hamstra et al., 2011; Fan et al., 2020), are more likely to perceive the demands and risks (e.g., responsibility, ambiguity) inherent in empowerment. This threat-sensitive appraisal makes them more susceptible to the health-impairment pathway, strengthening the link between empowering leadership and role stress (Bakker and Demerouti, 2017; Dennerlein and Kirkman, 2022).

*H3a*: Promotion focus moderates the relationship between empowering leadership and work passion, such that the relationship is stronger when promotion focus is high.

*H3b*: Prevention focus moderates the relationship between empowering leadership and role stress, such that the relationship is stronger when prevention focus is high.

*H3c*: The indirect effect of empowering leadership on illegitimate task perceptions via work passion is moderated by promotion focus, being stronger when promotion focus is high.

*H3d*: The indirect effect of empowering leadership on illegitimate task perceptions via role stress is moderated by prevention focus, being stronger when prevention focus is high.

*H3*: Regulatory focus moderates the indirect effects of empowering leadership on illegitimate task perceptions via (a) work passion and (b) role stress. Specifically, the indirect effect via work passion is stronger for employees high (vs. low) in promotion focus. The indirect effect via role stress is stronger for employees high (vs. low) in prevention focus.

## 3 Methods

### 3.1 Sample and procedure

This cross-sectional study collected data from full-time corporate employees in China using anonymous online questionnaires administered via the Credamo and Wenjuanxing platforms. Before completing the survey, participants were informed that participation was voluntary, responses would remain confidential, and the data would be used solely for academic purposes. Ethical approval was obtained prior to data collection, and all participants provided written informed consent.

After removing incomplete responses, 354 valid questionnaires were retained, resulting in an 86.76% retention rate. To mitigate common method bias, survey items were deliberately labeled with neutral terms to obscure the actual constructs (e.g., ‘work passion’ and ‘role stress’ were masked). The sample comprised 48.9% male and 51.1% female respondents, with 79.9% aged 20–39 years. Most participants held bachelor’s degrees or higher (76.5%), possessed over 1 year of work experience, and over half reported 3–10 years of tenure with their current supervisor. The sample included employees from various organizational types: state-owned enterprises (25.4%), private firms (19.2%), foreign-invested enterprises (11.9%), government agencies (33.6%), and public institutions (9.9%), providing broad coverage across key sectors of the Chinese economy.

### 3.2 Measurement

All scales used were established, validated instruments, ensuring alignment between organizational phenomena, research questions, conceptual definitions, and measurement. Responses were recorded on a 5-point Likert scale (1 = “Strongly Disagree,” 5 = “Strongly Agree”).

#### 3.2.1 Empowering leadership

Measured using Ahearne et al.’s (2005) 12-item scale, encompassing four dimensions: meaning of work, participation in decision-making, confidence in high performance, and work autonomy. Sample item: “My leader has confidence in my ability to perform my job well.”

#### 3.2.2 Perceptiong of illegitimate tasks

Assessed with the 8-item scale by Semmer et al. (2010), adapted for the Chinese context by Ge et al. (2022). It comprises two dimensions: unreasonable tasks and unnecessary tasks. Sample item: “I have to deal with tasks that are completely meaningless.”

#### 3.2.3 Work passion

Measured using the Harmonious Passion subscale (7 items) from Vallerand et al.'s (2003) Passion Scale, selected to capture the internalization of work autonomy. Sample item: “My work is in harmony with other activities in my life.”

#### 3.2.4 Role stress

Assessed using Peterson et al.'s (1995) 13-item scale, revised by Li and Zhang (2009) for Chinese organizations. It measures three dimensions: role conflict, role ambiguity, and role overload. Sample item: “I have too much work to do everything well.”

#### 3.2.5 Promotion focus and prevention focus

Measured using Lockwood et al.'s (2002) scale adapted by Zhou et al. (2012), featuring promotion focus (4 items) and prevention focus (3 items). Sample items: “I typically focus on achieving positive outcomes in the future” (Promotion); “I often worry I will not fulfill my work obligations” (Prevention).

#### 3.2.6 Control variables

To rule out potential confounding effects, six variables were set as controls, referring to prior studies on illegitimate task perceptions and leadership effectiveness (Semmer et al., 2015; Li et al., 2024): gender, age, education level, organization type, job tenure, and tenure with supervisor.

## 4 Results

### 4.1 Common method bias test

Despite implementing procedural controls (e.g., anonymous surveys, item obscuring) to mitigate common method variance (CMV) in this single-wave survey, residual effects cannot be entirely ruled out. We therefore conducted Harman’s single-factor test. The unrotated factor solution yielded eight factors with eigenvalues >1. The first factor accounted for 22.61% of the variance, below the critical threshold of 40%. This indicates no serious CMV issues in the data.

### 4.2 Confirmatory factor analyses

Confirmatory factor analysis (CFA) was conducted for all study variables. To assess discriminant validity, we compared the hypothesized six-factor measurement model with a series of alternative models in which conceptually related constructs were combined: a five-factor model (role stress and promotion focus combined); a four-factor model (role stress with promotion focus, and prevention focus with perceptions of illegitimate tasks, combined); a three-factor model (empowering leadership with work passion; role stress with promotion focus; and prevention focus with perceptions of illegitimate tasks, combined); a two-factor model (empowering leadership with work passion combined, and role stress, promotion focus, prevention focus, and perceptions of illegitimate tasks combined); and a one-factor model (all constructs combined). As shown in Table 1, the hypothesized six-factor model (*χ*^2^ = 628.19, df = 335; *χ*^2^/df = 1.88, RMSEA = 0.05, CFI = 0.96, TLI = 0.95, IFI = 0.96) fitted the data substantially better than the alternative models, supporting the distinctiveness of the six constructs.

**Table 1:** Confirmatory factor analyses.

Model	*c^2^*	*df*	*c^2^/df*	*RMSEA*	*CFI*	*TLI*	*IFI*
Baseline model	628.19	335	1.88	0.05	0.96	0.95	0.96
Five-factor model	1497.92	340	4.41	0.10	0.84	0.82	0.84
Four-factor model	2024.46	344	5.89	0.12	0.77	0.74	0.77
Three-factor model	2558.88	347	7.37	0.13	0.69	0.67	0.69
Two-factor model	3406.55	349	9.76	0.16	0.58	0.54	0.58
One-factor model	4613.98	350	13.18	0.19	0.41	0.36	0.41

In addition, Table 2 reports internal consistency and convergent validity indices. Cronbach’s *α* ranged from 0.87 to 0.94, and composite reliability (CR) ranged from 0.88 to 0.94, indicating good reliability. Average variance extracted (AVE) values ranged from 0.50 to 0.70 for all constructs; role stress was marginally below the conventional 0.50 threshold (AVE = 0.49) but demonstrated high reliability (α = 0.93; CR = 0.92), suggesting acceptable convergent validity overall.

**Table 2:** Reliability and convergent validity of the constructs.

Construct	No. of items	Cronbach’s α	Composite reliability (CR)	Average variance extracted (AVE)
Empowering leadership	12	0.92	0.92	0.50
Work passion	7	0.92	0.92	0.62
Role stress	13	0.93	0.92	0.49
Promotion focus	4	0.88	0.89	0.66
Prevention focus	3	0.87	0.88	0.70
Illegitimate task perception	8	0.94	0.94	0.66

### 4.3 Descriptive statistics and correlations

Descriptive statistics and correlations are presented in Table 3. Empowering leadership showed a significant positive correlation with work passion (*r* = 0.32**, *p* < 0.01) and role stress (*r* = 0.25**, *p* < 0.01); Work passion correlated negatively with illegitimate task perceptions (*r* = −0.35**, *p* < 0.01), while role stress correlated positively (*r* = 0.19**, *p* < 0.01). These results align with the hypothesized relationships.

**Table 3:** Descriptive statistics and correlations.

Variables	*M*	*SD*	1	2	3	4	5	6	7	8	9	10	11	12
1. Gender	1.51	0.50												
2. Age	2.91	0.88	−0.04											
3. Education level	3.92	0.84	0.02	−0.11^*^										
4. Organization type	2.83	1.39	−0.01	−0.03	0.05									
5. Job tenure	3.59	1.14	−0.09	0.69^**^	0.02	0.02								
6. Tenure with supervisor	2.93	1.15	−0.06	0.71^**^	0.01	0.04	0.76^**^							
7. Empowering leadership	3.73	0.69	−0.02	−0.01	0.05	0.04	0.15^**^	0.13^*^	(0.92)					
8. Promotion focus	3.70	0.88	0.18^**^	0.07	0.12^*^	−0.02	0.04	0.03	0.11^*^	(0.88)				
9. Prevention focus	3.02	1.11	−0.09	−0.06	−0.22^**^	0.02	−0.13^*^	−0.04	−0.13^*^	−0.36^**^	(0.87)			
10. Work passion	3.65	0.84	0.11^*^	0.15^**^	0.14^**^	0.03	0.15^**^	0.15^**^	0.32^**^	0.54^**^	−0.28^**^	(0.92)		
11. Role stress	3.24	0.81	−0.09	−0.01	−0.03	0.02	0.01	0.09	0.25^**^	−0.23^**^	0.37^**^	−0.07	(0.93)	
12. Perception of illegitimate tasks	2.76	0.93	−0.10	−0.04	−0.12^*^	0.03	−0.08	−0.07	−0.26^**^	−0.42^**^	0.38^**^	−0.35^**^	0.19^**^	(0.94)

*N* = 354; *p < 0.05; **p < 0.01; ***p < 0.001; Values in parentheses represent Cronbach’s alpha coefficients.

### 4.4 Hypotheses testing

Hierarchical regression analyzes were conducted to test the proposed hypotheses. As shown in Model M2 (Table 4), empowering leadership was positively associated with work passion (*b* = 0.39, *p* < 0.001) after controlling for demographic variables. Model M9 further indicated that work passion was negatively related to perceptions of illegitimate tasks (*b* = −0.37, *p* < 0.001). In parallel, Model M5 showed that empowering leadership was positively associated with role stress (*b* = 0.30, *p* < 0.001), and Model M10 demonstrated that role stress was positively associated with perceptions of illegitimate tasks (*b* = 0.22, *p* < 0.001). Taken together, these results support H1a, H1b, H2a and H2b.

**Table 4:** Regression-based mediation analysis 1.

Variables	WP			RS			PIT				
	M1	M2	M3	M4	M5	M6	M7	M8	M9	M10	M11
Gender	0.11	0.10	0.03	−0.09	−0.10	−0.06	−0.16	−0.15	−0.12	−0.14	−0.10
Age	0.11	0.19^*^	0.12	−0.11	−0.05	−0.02	0.02	−0.06	0.05	0.04	0.01
Education level	0.16^**^	0.15^**^	0.09^*^	−0.04	−0.04	0.05	−0.13^*^	−0.13^*^	−0.08	−0.13^*^	−0.08
Organization type	0.01	0.01	0.01	0.01	0.00	0.00	0.03	0.03	0.03	0.03	0.03
Tenure with supervisor	0.03	−0.01	0.02	0.17^**^	0.15^*^	0.10	−0.04	−0.01	−0.03	−0.07	−0.05
Job tenure	0.03	−0.02	−0.02	−0.07	−0.11	−0.05	−0.05	−0.01	−0.04	−0.04	0.02
EL		0.39^***^	0.34^***^		0.30^***^	0.33^***^		−0.34^***^			−0.32^***^
PROF			0.45^***^								
PREF						0.26^***^					
EL × PROF			0.15^**^								
EL × RREF						0.18^**^					
WP									−0.37^***^		−0.26^***^
RS										0.22^***^	0.27^***^
*R*^^2^	0.05	0.15	0.40	0.03	0.09	0.27	0.03	0.09	0.13	0.07	0.21
Adj. *R*^^2^	0.04	0.13	0.38	0.01	0.07	0.25	0.01	0.07	0.12	0.05	0.19
*F*	3.29^**^	8.86^***^	25.44^***^	1.59	4.71^***^	13.99^***^	1.75	4.98^***^	7.63^***^	3.46^**^	9.91^***^

*N* = 354; *p < 0.05; **p < 0.01; ***p < 0.001.

Subsequent mediation analyzes revealed that empowering leadership had a significant negative direct association with perceptions of illegitimate tasks (Model M8: *b* = −0.34, *p* < 0.001) after accounting for demographic variables. When both mediators were entered simultaneously (Model M11), work passion remained a significant negative predictor (*b* = −0.26, *p* < 0.001), whereas role stress remained a significant positive predictor (*b* = 0.27, *p* < 0.001), providing evidence of parallel mediation. The direct effect of empowering leadership also remained significant (*b* = −0.32, *p* < 0.001), suggesting partial mediation. Thus, H1 and H2 were supported.

To examine the moderating effects of regulatory focus, empowering leadership and regulatory focus variables were mean-centered prior to creating the interaction terms. As shown in Model M3, the interaction between empowering leadership and promotion focus was positively associated with work passion (*b* = 0.15, *p* < 0.001), supporting the moderating role of promotion focus in the empowering leadership–work passion relationship. Similarly, Model M6 indicated that the empowering leadership × prevention focus interaction was positively associated with role stress (*b* = 0.18, *p* < 0.001), supporting the moderating role of prevention focus in the empowering leadership–role stress relationship.

**Figure 2:**
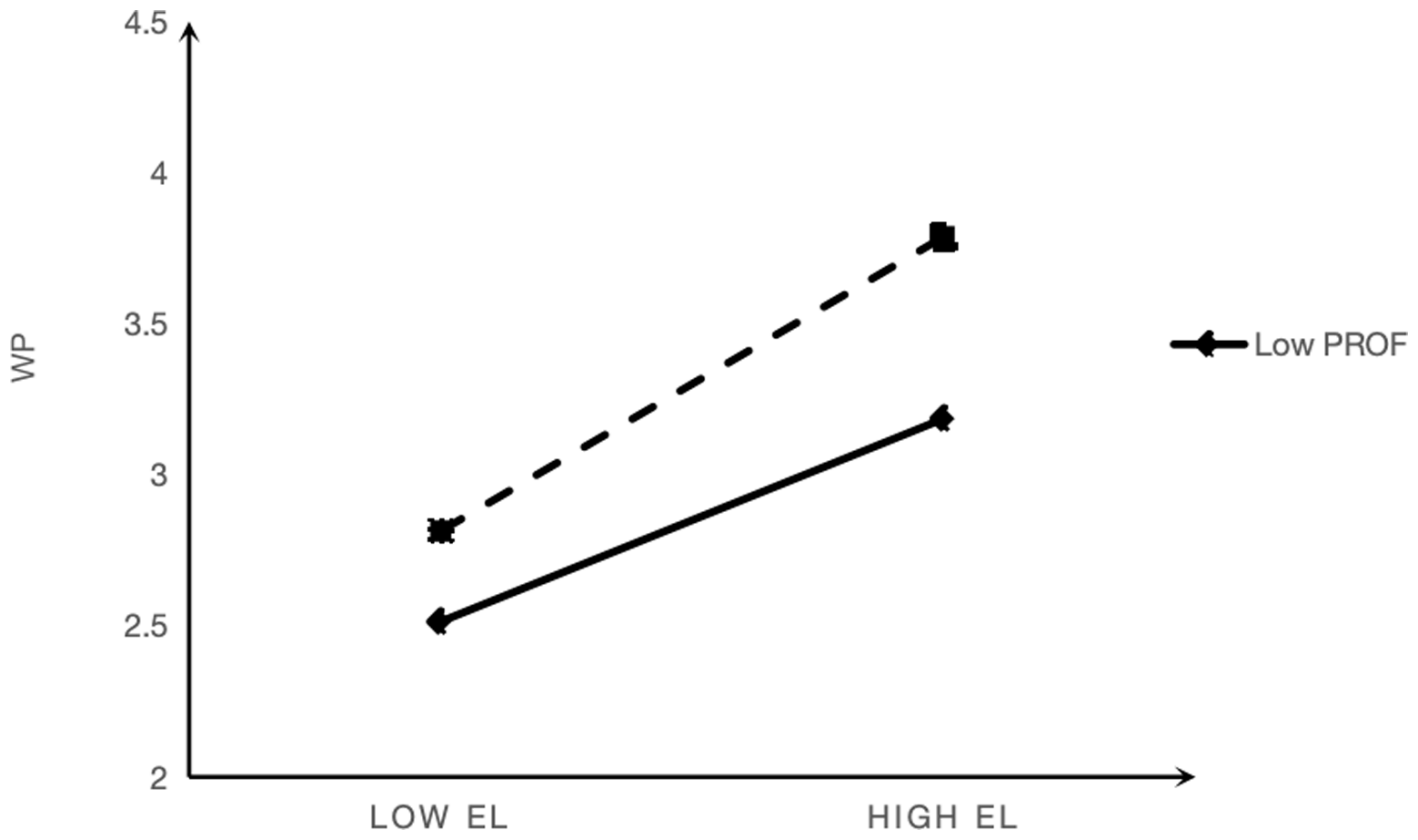
Promotion focus as a moderator of empowering leadership and work passion.

**Figure 3:**
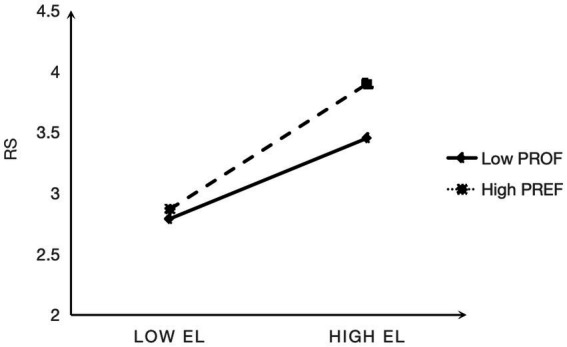
Prevention focus as a moderator of empowering leadership and role stress.

To facilitate interpretation, simple slope plots were generated at the mean ± 1 SD. Figure 2 shows that the positive association between empowering leadership and work passion was stronger when employees reported higher (vs lower) promotion focus. Figure 3 shows that the positive association between empowering leadership and role stress was stronger under high (vs low) prevention focus. These patterns support H3a and H3b.

Moderated mediation was further tested using bootstrapping. As reported in Table 5, at low promotion focus, the indirect effect of empowering leadership on perceptions of illegitimate tasks via work passion was significant (effect = −0.06, 95% CI [−0.13, −0.01]). At high promotion focus, this indirect effect remained significant and was stronger (effect = −0.14, 95% CI [−0.22, −0.08]). The difference between the two conditional indirect effects was significant (effect = −0.08, 95% CI [−0.17, −0.02]), indicating that promotion focus strengthens the indirect effect via work passion. Therefore, Hypothesis 3c was supported.

**Table 5:** Test of moderated mediation.

Path	PROF	Effect	Bootstrap SE	Bootstrap LLCI	Bootstrap ULCI
The mediating effect of WP	Low (M − 1SD)	−0.06	0.03	−0.13	−0.01
	M	−0.10	0.03	−0.17	−0.05
	High (M + 1SD)	−0.14	0.04	−0.22	−0.08
	Differences (high-low)	−0.08	0.04	−0.17	−0.02
The mediating effect of RS	Low (M − 1SD)	0.04	0.03	−0.02	0.11
	M	0.10	0.03	0.06	0.16
	High (M + 1SD)	0.17	0.04	0.09	0.24
	Differences (high-low)	0.13	0.05	0.04	0.22

The second conditional indirect effect is also reported in Table 5. At high prevention focus, the indirect effect of empowering leadership on perceptions of illegitimate tasks via role stress was significant (effect = 0.17, 95% CI [0.09, 0.24]), whereas at low prevention focus it was not significant (effect = 0.04, 95% CI [−0.02, 0.11]). The difference between the two conditional indirect effects was significant (effect = 0.13, 95% CI [0.04, 0.22]), indicating that prevention focus strengthens the indirect effect via role stress. Therefore, Hypothesis 3d was supported.

## 5 Discussion

Grounded in an integrated framework that places Role Theory at its core, this study examines empowering leadership not only as a source of resources or demands but as a key catalyst for role boundary redefinition. By integrating the Job Demands–Resources (JD-R) model (Bakker and Demerouti, 2017), we develop and test a moderated parallel mediation model to explain how and why empowering leadership can lead to divergent perceptions of illegitimate tasks. Our findings highlight the psychological processes through which employees cognitively evaluate and respond to changes in their role boundaries initiated by leadership (Katz and Kahn, 1978; Semmer et al., 2010). We also emphasize that employees’ regulatory focus (Higgins, 1997; Yan et al., 2024), whether promotion-focused or prevention-focused, significantly shapes how they perceive and respond to role redefinition, leading to distinct cognitive evaluations of illegitimate tasks.

### 5.1 Key findings and theoretical interpretation

Our findings confirm that empowering leadership exerts a ‘double-edged sword’ effect on illegitimate task perception via two opposing psychological pathways (Dennerlein and Kirkman, 2022; Rai et al., 2024). This duality originates from the fundamental character of empowering leadership as a formal proposal for role redefinition (Ahearne et al., 2005). When presented with this proposal, employees undertake a cognitive appraisal that may result in either role integration or role exclusion.

On the one hand, the integration path operates through work passion. Empowering leadership supplies critical resources such as autonomy, trust, and meaningfulness (Bakker and Demerouti, 2017), which fulfill employees’ psychological needs and foster a strong, identity-internalized work passion (Vallerand et al., 2003; Egan et al., 2019; Ho et al., 2018). Employees with heightened passion are more inclined to proactively incorporate newly delegated tasks into their existing role identity. This cognitive integration reduces the perceived discrepancy between the task and their role boundaries, thereby diminishing perceptions of illegitimacy (Semmer et al., 2015). This pathway aligns with the motivational process of the JD-R model and constitutes a successful assimilation of the role redefinition proposal (Bakker and Demerouti, 2007; Lee and Jo, 2023).

On the other hand, the same leadership behaviors instigate an exclusion path via role stress. By introducing new demands, such as heightened responsibility, complexity, and ambiguity (Lorinkova et al., 2013), empowering leadership can trigger role stress, which is characterized by ambiguity, overload, and conflict (Kahn et al., 1964; Thun et al., 2018; Tang et al., 2023). Under such stress, employees tend to adopt a defensive posture toward their role boundaries (Hobfoll, 1989). To conserve resources and preserve a manageable role schema, they are more likely to cognitively reject new tasks by categorizing them as transgressions of their professional domain, thus perceiving them as illegitimate (Semmer et al., 2010; Ahmed et al., 2018; Meier and Semmer, 2018). This pathway corresponds to the health impairment process of the JD-R model and represents a defensive rejection of the role redefinition proposal.

Second, our study clarifies a pivotal boundary condition: an individual’s regulatory focus (Higgins, 1997). This cognitive tendency serves as the primary ‘lens’ through which the role redefinition proposal is evaluated (Hamstra et al., 2011; Lanaj et al., 2012). For promotion-focused individuals, oriented toward growth and achievement, the proposal is filtered as an opportunity; they are more attuned to the resources provided, which amplifies the link between empowering leadership and work passion, thereby strengthening the integration path. For prevention-focused individuals, oriented toward safety and obligation, the proposal is filtered as a threat; they are more sensitive to the demands and risks involved, which intensifies the link between empowering leadership and role stress, thereby strengthening the exclusion path (Fan et al., 2020). Thus, regulatory focus importantly shapes which cognitive-evaluative route—integration or exclusion—becomes more salient in the role redefinition context initiated by the leader.

### 5.2 Theoretical implications

This research makes three primary theoretical contributions by shifting the perspective on illegitimate tasks toward role definition and appraisal.

First, this study advances the application of role theory to explain the antecedents of illegitimate tasks. Prior research has often treated role boundaries as a relatively static backdrop to task conflict (Ma and Peng, 2019; Fältén et al., 2024). Our work theorizes and empirically validates leader-induced role boundary redefinition as a critical antecedent (Zhou et al., 2020). We move beyond asking “what tasks are illegitimate” to explore “how tasks come to be perceived as illegitimate” through the cognitive appraisal of a leader’s redefinition proposal. This positions illegitimate task perception within a framework of role cognition (Meier and Semmer, 2018).

Second, by integrating the JD-R model (Bakker and Demerouti, 2017) into the role redefinition framework, this research provides a coherent mechanism for empowering leadership’s double-edged sword effect. Whereas previous studies have often examined its positive and negative outcomes in isolation (Kim and Beehr, 2018; S. Lee et al., 2017; Dennerlein and Kirkman, 2022; Rai et al., 2024; Wang et al., 2021; Ye et al., 2022), we demonstrate that work passion and role stress are not merely parallel mediators but are the concrete psychological embodiments of the integration and exclusion processes, respectively. This clarifies how the same leadership behavior simultaneously triggers opposing motivational and strain pathways that competitively shape the final judgment of task legitimacy (Han et al., 2019), offering a more integrative understanding of leadership complexity.

Third, by introducing regulatory focus as a key individual difference (Higgins, 1997; Higgins and Pinelli, 2020), we specify the boundary conditions of the model, answering the question: “For whom is role redefinition more likely an opportunity for integration, and for whom is it a trigger for defensive exclusion?” Our findings show that employees’ chronic motivational dispositions systematically shape their primary appraisal of the redefinition proposal (Lanaj et al., 2012; Fan et al., 2020), channeling the subsequent psychological process along one path more than the other. This transforms the model from a universal framework into a more contingent theory that predicts which psychological pathway will be more salient for which type of employee, significantly enhancing its explanatory precision and practical relevance.

### 5.3 Practical implications

Our findings offer nuanced guidance for managers seeking to implement empowerment effectively without inadvertently heightening illegitimate task perceptions.

Managers should frame empowerment as a collaborative role redefinition, not a unilateral delegation. This involves explicitly communicating empowerment as a joint process of reviewing and adjusting role boundaries and growth opportunities, rather than merely assigning extra duties. Practically, this means discussing how new autonomy aligns with the employee’s career aspirations (promoting integration) while proactively clarifying expectations and providing necessary resources to minimize ambiguity (preventing exclusion) (Song and Chen, 2021).

To foster the integration path, leaders should couple empowerment with actions that fuel work passion, such as connecting tasks to personal meaning, recognizing initiative, and providing opportunities for skill development and mastery (Egan et al., 2019; Ho et al., 2018). To mitigate the exclusion path, organizations need to implement supportive structures, including clear guidelines to reduce role ambiguity, realistic workload assessments to prevent overload, and accessible mentoring to help employees navigate new complexities (Dennerlein and Kirkman, 2022).

Given the moderating role of regulatory focus, adopting personalized role communication strategies is essential. For promotion-focused employees, emphasize the growth, learning, and achievement potential inherent in the new role boundaries. For prevention-focused employees, focus communications on providing stability, clear procedures, risk mitigation plans, and reassurance about safety and competence within the redefined role (Higgins and Pinelli, 2020; Lanaj et al., 2012). This tailored approach makes it more likely the empowerment proposal will be appraised as an opportunity rather than a threat.

### 5.4 Limitations and future research

This study has several limitations that point to fruitful directions for future research.

First, the cross-sectional nature of the data precludes examination of the dynamic recalibration process at the heart of our role-redefinition framework. While the model elucidates associated psychological mechanisms, it cannot establish the temporal precedence of empowering leadership or track how subsequent cognitive appraisals and role boundaries evolve. Future longitudinal or experience-sampling research is essential to delineate how perceptions of role scope and task illegitimacy develop and stabilize over time following an empowering intervention (Kanat-Maymon et al., 2020).

Second, the primary reliance on self-report data carries the risk of common method bias, despite procedural precautions taken during data collection. Future studies could strengthen validity by incorporating multi-source data (e.g., leader ratings of empowering behavior) or employing experimental methods to manipulate key variables. Particularly in research on illegitimate tasks, situational experiments or critical incident techniques could be valuable for examining how specific leadership acts trigger legitimacy judgments (Meier and Semmer, 2018).

Third, the theoretical framework and analysis are situated at the individual level. Future work could explore a multi-level perspective. For instance, research could investigate how a team-level climate of empowering leadership influences individual outcomes through team-level processes like collective efficacy and shared passion. Building on multilevel evidence that individual and collective states jointly shape work outcomes (Martinaityte et al., 2020), cross-level studies examining the interaction between individual differences and team climate could also yield valuable insights.

Finally, the study was conducted within Chinese organizations, which are characterized by a high power-distance cultural context. In such settings, employees typically exhibit a higher degree of deference to authority, which may make them more receptive to leadership-driven changes in role boundaries. However, for individuals who prefer stability or are more resistant to change, the conflict between their expectations of formal role boundaries and the ambiguity introduced by empowerment could lead to heightened perceptions of illegitimate tasks. Future cross-cultural comparative research could test the model’s applicability across different cultural dimensions, such as power distance and uncertainty avoidance, to better understand its generalizability and cultural boundaries (Hofstede, 2001).

## Data Availability

The raw data supporting the conclusions of this article will be made available by the authors, without undue reservation.

## References

[ref1] Ahearne M. Mathieu J. Rapp A. (2005). To empower or not to empower your sales force? An empirical examination of the influence of leadership empowerment behavior on customer satisfaction and performance. J. Appl. Psychol. 90, 945–955. doi: 10.1037/0021-9010.90.5.945, 16162066

[ref2] Ahmed S. F. Eatough E. M. Ford M. T. (2018). Relationships between illegitimate tasks and change in work-family outcomes via interactional justice and negative emotions. J. Vocat. Behav. 104, 14–30. doi: 10.1016/j.jvb.2017.10.002

[ref3] Arshad M. Qasim N. Farooq O. Rice J. (2022). Empowering leadership and employees’ work engagement: a social identity theory perspective. Manage. Decis. 60, 1218–1236. doi: 10.1108/MD-11-2020-1485

[ref4] Bakker A. B. Demerouti E. (2007). The job demands–resources model: state of the art. J. Manag. Psychol. 22, 309–328. doi: 10.1108/02683940710733115

[ref5] Bakker A. B. Demerouti E. (2017). Job demands–resources theory: taking stock and looking forward. J. Occup. Health Psychol. 22, 273–285. doi: 10.1037/ocp0000056, 27732008

[ref6] Bakker A. B. Demerouti E. Euwema M. C. (2005). Job resources buffer the impact of job demands on burnout. J. Occup. Health Psychol. 10, 170–180. doi: 10.1037/1076-8998.10.2.170, 15826226

[ref9002] Deci E. L. Ryan R. M. (2000). The “what” and “why” of goal pursuits: Human needs and the self-determination of behavior. Psychological Inquiry, 11, 227–268. doi: 10.1207/S15327965PLI1104_01

[ref7] Dennerlein T. Kirkman B. L. (2022). The hidden dark side of empowering leadership: the moderating role of hindrance stressors in explaining when empowering employees can promote moral disengagement and unethical pro-Organisational behavior. J. Appl. Psychol. 107, 2220–2242. doi: 10.1037/apl0001013, 35286112

[ref8] Eatough E. M. Meier L. L. Igic I. Elfering A. Spector P. E. Semmer N. K. (2016). You want me to do what? Two daily diary studies of illegitimate tasks and employee well-being. J. Organ. Behav. 37, 108–127. doi: 10.1002/job.2032

[ref9] Egan R. Zigarmi D. Richardson A. (2019). Leadership behavior: a partial test of the employee work passion model. Hum. Resour. Dev. Q. 30, 311–341. doi: 10.1002/hrdq.21346

[ref10] Fältén R. Berntson E. Bernhard-Oettel C. (2024). How are organisational conditions related to illegitimate tasks among managers and their subordinates in the public sector? A Swedish study. Work Stress. 38, 270–292. doi: 10.1080/02678373.2024.2356646

[ref11] Fan X. Wang Q. Liu J. Liu C. Cai T. (2020). Why do supervisors abuse subordinates? Effects of team performance, regulatory focus, and emotional exhaustion. J. Occup. Organ. Psychol. 93, 605–628. doi: 10.1111/joop.12307

[ref12] García Johnson C. P. Otto K. (2020). “Please, bring me some coffee”: illegitimate tasks as the explanation for the relationship between Organisational sexism and occupational well-being. GENDER – Z. Geschl. Kult. Ges. 12, 124–140. doi: 10.3224/gender.v12i3.09, 40176700

[ref13] Ge W. Wu P. Zeng L. Yafu H. Quartz (2022). Revision of the illegitimate task scale in a Chinese employee sample. Chin. J. Clin. Psychol. 30, 336–339. doi: 10.16128/j.cnki.1005-3611.2022.02.018

[ref14] Hamstra M. R. W. Van Yperen N. W. Wisse B. Sassenberg K. (2011). Transformational–transactional leadership styles and followers’ regulatory focus: fit reduces followers’ turnover intentions. J. Pers. Psychol. 10, 182–186. doi: 10.1027/1866-5888/a000043

[ref15] Han S. Harold C. M. Cheong M. (2019). Examining why employee proactive personality influences empowering leadership: the roles of cognition- and affect-based trust. J. Occup. Organ. Psychol. 92, 352–383. doi: 10.1111/joop.12252

[ref16] Higgins E. T. (1997). Beyond pleasure and pain. Am. Psychol. 52, 1280–1300. doi: 10.1037/0003-066X.52.12.1280, 9414606

[ref17] Higgins E. T. Pinelli F. (2020). Regulatory focus and fit effects in organizations. Annu. Rev. Organ. Psychol. Organ. Behav. 7, 25–48. doi: 10.1146/annurev-orgpsych-012119-045404

[ref18] Ho V. T. Kong D. T. Lee C.-H. Dubreuil P. Forest J. (2018). Promoting harmonious work passion among unmotivated employees: a two-nation investigation of the compensatory function of cooperative psychological climate. J. Vocat. Behav. 106, 112–125. doi: 10.1016/j.jvb.2018.01.005

[ref19] Hobfoll S. E. (1989). Conservation of resources: a new attempt at conceptualizing stress. Am. Psychol. 44, 513–524. doi: 10.1037/0003-066X.44.3.513, 2648906

[ref20] Hofstede G. (2001). Culture’s consequences: Comparing values, behaviors, institutions and organisations across nations. 2nd Edn. Thousand Oaks, CA: Sage Publications.

[ref21] Jacobshagen N. (2006). Illegitimate tasks, illegitimate stressors: Testing a new stressor–strain concept. [doctoral dissertation]. Bern: University of Bern.

[ref22] Jin J. Xu S. Wang Y. (2014). A comparison study of role overload, work-family conflict and depression between China and North America: the moderation effect of social support. Acta Psychol. Sin. 46, 1144–1160. doi: 10.3724/SP.J.1041.2014.01144

[ref9001] Kahn R. L. Wolfe D. M. Quinn R. P. Snoek J. D Rosenthal R. A. (1964). Organizational Stress: Studies in Role Conflict and Ambiguity. New York: Wiley.

[ref23] Kanat-Maymon Y. Elimelech M. Roth G. (2020). Work motivations as antecedents and outcomes of leadership: integrating self-determination theory and the full range leadership theory. Eur. Manag. J. 38, 555–564. doi: 10.1016/j.emj.2020.01.003

[ref24] Katz D. Kahn R. L. (1978). The social psychology of organisations. 2nd Edn. New York: Wiley.

[ref25] Khumalo N. Olaleye B. R. (2025). Emotional demands and role ambiguity influence on intentions to quit: does trust in management matter? Admin. Sci. 15:424. doi: 10.3390/admsci15110424

[ref26] Kim M. Beehr T. A. (2018). Can empowering leaders affect subordinates’ well-being and careers because they encourage subordinates’ job crafting behaviors? J. Leadersh. Organ. Stud. 25, 184–196. doi: 10.1177/1548051817727702

[ref27] Lanaj K. Chang C.-H. D. Johnson R. E. (2012). Regulatory focus and work-related outcomes: a review and Meta-analysis. Psychol. Bull. 138, 998–1034. doi: 10.1037/a0027723, 22468880

[ref28] Lee S. Cheong M. Kim M. Yun S. (2017). Never too much? The curvilinear relationship between empowering leadership and task performance. Group Organ. Manag. 42, 11–38. doi: 10.1177/1059601116646474

[ref29] Lee D. Y. Jo Y. (2023). The job demands–resource model and performance: the mediating role of employee engagement. Front. Psychol. 14:1194018. doi: 10.3389/fpsyg.2023.1194018, 37425190 PMC10323440

[ref30] Lee A. Willis S. Tian A. W. (2018). Empowering leadership: a meta-analytic examination of incremental contribution, mediation, and moderation. J. Organ. Behav. 39, 306–325. doi: 10.1002/job.2220

[ref31] Li W. Xv Z. Ji B. (2024). How empowering leadership can improve new employee engagement: the mediating role of employee perceptions of insider status. SAGE Open 14, 1–11. doi: 10.1177/21582440241271243

[ref32] Li C. P. Zhang Y. (2009). The impact of role stressors on teachers’ physical and mental health. Psychol. Dev. Educ. 25, 114–119. doi: 10.16187/j.cnki.issn1001-4918.2009.01.017

[ref33] Lockwood P. Jordan C. H. Kunda Z. (2002). Motivation by positive or negative role models: regulatory focus determines who will best inspire us. J. Pers. Soc. Psychol. 83, 854–864. doi: 10.1037/0022-3514.83.4.854, 12374440

[ref34] Lorinkova N. M. Pearsall M. J. Sims H. P. (2013). Examining the differential longitudinal performance of directive versus empowering leadership in teams. Acad. Manag. J. 56, 573–596. doi: 10.5465/amj.2011.0132

[ref35] Ma J. Peng Y. (2019). The performance costs of illegitimate tasks: the role of job identity and flexible role orientation. J. Vocat. Behav. 110, 144–154. doi: 10.1016/j.jvb.2018.11.012

[ref36] Martinaityte I. Unsworth K. L. Sacramento C. A. (2020). Is the project ‘mine’ or ‘ours’? A multilevel investigation of the effects of individual and collective psychological ownership. J. Occup. Organ. Psychol. 93, 302–327. doi: 10.1111/joop.12300

[ref37] Meier L. L. Semmer N. K. (2018). Illegitimate tasks as assessed by incumbents and supervisors: converging only modestly but predicting strain as assessed by incumbents, supervisors, and partners. Eur. J. Work Organ. Psychol. 27, 403–419. doi: 10.1080/1359432X.2018.1526785

[ref38] Morrison E. W. (1994). Role definitions and organizational citizenship behavior: the importance of the employee's perspective. Acad. Manag. J. 37, 1543–1567. doi: 10.5465/256798

[ref39] Paterson T. A. Huang L. (2018). Am i expected to be ethical? A role-definition perspective of ethical leadership and unethical behavior. J. Manage. 45, 2837–2860. doi: 10.1177/0149206318771166

[ref40] Peng J. Wang Z. (2018). Being a prototypic follower: burdening or enabling? The paradoxical effect of followership prototype-trait match. Acta Psychol. Sin. 50, 216–229. doi: 10.3724/SP.J.1041.2018.00216, 41207781

[ref41] Perrewé P. L. Hochwarter W. A. Ferris G. R. McAllister C. P. Harris J. N. (2014). Developing a passion for work passion: future directions on an emerging construct. J. Organ. Behav. 35, 145–150. doi: 10.1002/job.1902

[ref42] Peterson M. F. Smith P. B. Akande A. Ayestaran S. Bochner S. Callan V. . (1995). Role conflict, ambiguity, and overload: a 21-nation study. Acad. Manag. J. 38, 429–452. doi: 10.2307/256687

[ref43] Rai A. Kim M. Shukla A. (2024). A double-edged sword: empowering leadership to employees’ work-life interface. Int. J. Hum. Resour. Manage. 35, 3525–3555. doi: 10.1080/09585192.2024.2421348

[ref44] Rizzo J. R. House R. J. Lirtzman S. I. (1970). Role conflict and ambiguity in complex organizations. Adm. Sci. Q. 15, 150–163. doi: 10.2307/2391486

[ref45] Roy D. F. Kahn R. L. Wolfe D. M. Quinn R. P. Snoek J. D. Rosenthal R. A. (1965). Organisational stress: Studies in role conflict and ambiguity. New York: John Wiley & Sons.

[ref46] Semmer N. K. Jacobshagen N. Meier L. L. Elfering A. (2007). “Occupational stress research: the stress-as-offense-to-self perspective” in Occupational Health Psychology: European perspectives on research, education and practice. eds. Houdmont J. McIntyre S. (Castelo da Maia: ISMAI), 43–60.

[ref47] Semmer N. K. Jacobshagen N. Meier L. L. Elfering A. Beehr T. A. Kälin W. . (2015). Illegitimate tasks as a source of work stress. Work Stress. 29, 32–56. doi: 10.1080/02678373.2014.1003996, 25892839 PMC4396521

[ref48] Semmer N. K. Tschan F. Jacobshagen N. Beehr T. A. Elfering A. Kälin W. . (2019). Stress as offense to self: a promising approach comes of age. Occup. Health Sci. 3, 205–238. doi: 10.1007/s41542-019-00041-5, 32647746 PMC7328775

[ref49] Semmer N. K. Tschan F. Meier L. L. Facchin S. Jacobshagen N. (2010). Illegitimate tasks and counterproductive work behavior. Appl. Psychol. 59, 70–96. doi: 10.1111/j.1464-0597.2009.00416.x

[ref50] Song Q. Chen Y. (2021). The impact of the fit between needed and received empowering leadership on followers’ job-related outcomes: the mediating role of emotional exhaustion. Acta Psychol. Sin. 53, 890–903. doi: 10.3724/SP.J.1041.2021.00890

[ref51] Tang Y. Wang Y. Zhou H. Wang J. Zhang R. Lu Q. (2023). The relationship between psychiatric nurses’ perceived organisational support and job burnout: mediating role of psychological capital. Front. Psychol. 14:1099687. doi: 10.3389/fpsyg.2023.1099687, 36895741 PMC9989200

[ref52] Thun S. Halsteinli V. Løvseth L. (2018). A study of unreasonable illegitimate tasks, administrative tasks, and sickness Presenteeism amongst Norwegian physicians: an everyday struggle? BMC Health Serv. Res. 18:407. doi: 10.1186/s12913-018-3229-0, 29871623 PMC5989409

[ref53] Vallerand R. J. Blanchard C. Mageau G. A. Koestner R. Ratelle C. Léonard M. . (2003). Les Passions de l’Âme: On Obsessive and Harmonious Passion. J. Pers. Soc. Psychol. 85, 756–767. doi: 10.1037/0022-3514.85.4.756, 14561128

[ref54] Wang S. Eva N. Newman A. Zhou H. (2021). A double-edged sword: the effects of ambidextrous leadership on follower innovative behaviors. Asia Pac. J. Manag. 38, 1305–1326. doi: 10.1007/s10490-020-09714-0

[ref55] Yan J. Ali M. Khan M. M. Shah S. H. H. Butt A. S. (2024). The effect of promotion regulatory focus on service performance. Serv. Ind. J. 44, 45–62. doi: 10.1080/02642069.2021.2003340, 41307611

[ref56] Ye P. Liu L. Tan J. (2022). Influence of leadership empowering behaviour on employee innovation behaviour: the moderating effect of personal development support. Front. Psychol. 13:1022377. doi: 10.3389/fpsyg.2022.1022377, 36600721 PMC9806223

[ref57] Zhou Z. E. Eatough E. M. Che X. X. (2020). Effect of illegitimate tasks on work-to-family conflict through psychological detachment: passive leadership as a moderator. J. Vocat. Behav. 121:103463. doi: 10.1016/j.jvb.2020.103463

[ref58] Zhou Q. Hirst G. Shipton H. (2012). Context matters: combined influence of participation and intellectual stimulation on the promotion focus–employee creativity relationship. J. Organ. Behav. 33, 894–909. doi: 10.1002/job.779

[ref59] Zou Y. Zhang H. Peng J. Nie Q. Wang Z. (2023). Change or procrastination? Employees’ differentiated responses to illegitimate tasks. Acta Psychol. Sin. 55, 1529–1541. doi: 10.3724/SP.J.1041.2023.01529

